# *GUCY2D*-Associated Leber Congenital Amaurosis: A Retrospective Natural History Study in Preparation for Trials of Novel Therapies

**DOI:** 10.1016/j.ajo.2019.10.019

**Published:** 2020-02

**Authors:** Zaina Bouzia, Michalis Georgiou, Sarah Hull, Anthony G. Robson, Kaoru Fujinami, Tryfon Rotsos, Nikolas Pontikos, Gavin Arno, Andrew R. Webster, Alison J. Hardcastle, Alessia Fiorentino, Michel Michaelides

**Affiliations:** a Moorfields Eye Hospital, London, United Kingdom; b UCL Institute of Ophthalmology, University College London, London, United Kingdom; c Laboratory of Visual Physiology, Division of Vision Research, National Institute of Sensory Organs, National Hospital Organization Tokyo Medical Center, Tokyo, Japan; d Department of Ophthalmology, Keio University School of Medicine, Tokyo, Japan; e First Division of Ophthalmology, National and Kapodistrian University of Athens, General Hospital of Athens, Athens, Greece; f UK Inherited Retinal Dystrophy Consortium, United Kingdom

## Abstract

**Purpose:**

To describe the natural history of Leber congenital amaurosis (LCA) associated with *GUCY2D* variants (*GUCY2D*-LCA) in a cohort of children and adults, in preparation for trials of novel therapies.

**Design:**

Retrospective case series.

**Methods:**

Participants: Patients with *GUCY2D*-LCA at a single referral center. Procedures: Review of clinical notes, retinal imaging including fundus autofluorescence (FAF) and optical coherence tomography (OCT), electroretinography (ERG), and molecular genetic testing. Main Outcome Measures: Demographic data, symptoms at presentation, visual acuity, evidence of progression, OCT and FAF findings, ERG assessment, and molecular genetics.

**Results:**

Twenty-one subjects with *GUCY2D*-LCA were included, with a mean follow-up ± standard deviation (SD) of 10 ± 11.85 years. Marked reduction in visual acuity (VA) and nystagmus was documented in all patients within the first 3 years of life. Fifty-seven percent (n = 12) exhibited photophobia and 38% (n = 8) had nyctalopia. VA was worse than hand motion in 71% of the patients (n = 15). Longitudinal assessment of VA showed stability in all patients, except 1 patient who experienced deterioration over a follow-up of 44 years. Hyperopia was reported in 13 of the 17 subjects (71%) with available refraction data. Eighteen subjects had either normal fundus appearance (n = 14) or a blond fundus (n = 3), while only 4 of the eldest subjects had mild retinal pigment epithelium (RPE) atrophy (mean, 49 years; range 40-54 years). OCT data were available for 11 subjects and 4 different grades of ellipsoid zone (EZ) integrity were identified: (1) continuous/intact EZ (n = 6), (2) focally disrupted EZ (n = 2), (3) focally disrupted with RPE changes (n = 2), and (4) diffuse EZ disruption with RPE changes (n = 1). All examined subjects had stable OCT findings over the long follow-up period. Full-field ERGs showed evidence of a severe cone-rod dystrophy in 5 of 6 patients and undetectable ERGs in 1 subject. Novel genotype-phenotype correlations are also reported.

**Conclusion:**

*GUCY2D*-LCA is a severe early-onset retinal dystrophy associated with very poor VA from birth. Despite the severely affected photoreceptor function, the relatively preserved photoreceptor structure based on EZ integrity until late in the disease in the majority of subjects suggests a wide therapeutic window for gene therapy trials.

## Highlights

• *GUCY2D*-associated Leber congenital amaurosis is a severe early-onset retinal dystrophy.
• There is severe cone and rod dysfunction but with preserved photoreceptor structure evident on optical coherence tomography.
• Stable natural history suggests a wide therapeutic window for intervention.

Leber congenital amaurosis (LCA) represents a leading cause of autosomal recessive blindness in children worldwide, affecting between 1 in 30,000 to 1 in 81,000 newborns annually.^1^, ^2^, ^3^ Clinically, LCA is characterized by severe visual impairment at birth or within the first months of life. Affected individuals commonly exhibit nystagmus, the oculodigital sign (eye poking), and extinguished or severely abnormal electroretinography (ERG).^2^^,^^4^^,^^5^ LCA accounts for the most severe form of inherited retinal disorders, and both clinically and genetically overlaps with early-onset severe retinal dystrophy (EOSRD), which comprises milder phenotypes.^1^^,^^4^ EOSRD presents after infancy and before the age of 5 years. Affected individuals usually have better residual visual function than in LCA and minimal ERG signals.^2^^,^^4^ LCA/EOSRD is associated with disease-causing variants in 26 genes to date.^2^^,^^6^ It has been reported that certain genes are more likely to be associated with LCA, such as *GUCY2D*, *CEP290*, *NMNAT1*, and *AIPL1*, while variants in other genes more frequently cause EOSRD, including *RPE65* and *RDH12*.^2^ The genetic variability of LCA and the rarity of the condition make detailed phenotyping in a substantial molecularly confirmed cohort of patients challenging.

*GUCY2D* variants commonly cause LCA/EOSRD, accounting for 10%-20% of all cases.^4^ Different sequence variants in *GUCY2D* are common causes of autosomal dominant (AD) cone dystrophy (COD) and cone-rod dystrophy (CORD).^2^^,^^7^, ^8^, ^9^ *GUCY2D* encodes the photoreceptor enzyme guanylate cyclase 2D (GC-E), which synthesizes the intracellular messenger of photoreceptor excitation, cGMP, and is regulated by intracellular Ca^2+^-sensor proteins named guanylate cyclase–activating proteins (GCAPs). To date there are 144 identified variants in *GUCY2D*, with the majority reported to cause LCA/EOSRD (127 variants, 88%) and only 13 reported to cause AD-COD or AD-CORD. The AD-COD/CORD variants are all located in exon 13 (around the amino acid position 838) affecting the GC-E dimerization domain. In contrast, the variants reported to cause LCA do not have a localization hot spot but are scattered along the full length of the gene.^7^ The biochemical effect of many of the variants has been described both in vitro and in animal models. LCA/EOSRD-causing variants usually show either reduced ability or complete inability to synthesize the intracellular messenger cGMP.^10^, ^11^, ^12^ Moreover, some LCA/EOSRD-causing variants result in misfolding and consequent degradation of the protein in the endoplasmic reticulum.^12^ In contrast, COD/CORD-causing variants are functional but cause a shift in Ca^2+^ sensitivity.^7^ Despite the rather well-characterized genetic background of *GUCY2D*-LCA/EOSRD, the number of detailed phenotyping studies is limited.

Previous phenotyping studies identified evidence of preserved photoreceptor structure, in contrast to the severely affected functional findings of *GUCY2D*-LCA/EOSRD.^13^, ^14^, ^15^ Reduced visual acuity is a life-long source of morbidity for patients with LCA/EOSRD, with visual impairment having been significantly associated with increased risk of mortality.^16^ Gene-based approaches to therapy are used increasingly in clinical trials, with the first Food and Drug Administration–approved gene therapy for *RPE65*-LCA now available. Gene replacement therapy for *GUCY2D*-LCA/EOSRD has been investigated in animal studies, with considerable reported therapeutic success, using a range of vectors including recombinant adeno-associated virus serotype 2/8 (AAV2/8), adeno-associated virus serotype 5 (AAV5), and HIV1-based lentiviral vector.^2^^,^^17^^,^^18^ Aguirre and associates report an intact postgeniculate white matter pathway in subjects with *GUCY2D*-LCA/EOSRD, which provides further encouragement for the prospect of recovery of visual function with gene augmentation therapy.^19^ Jacobson and associates investigated potential outcome measures such as chromatic full-field sensitivity testing and optical coherence tomography (OCT), used to assess photoreceptor function and structure, respectively, concluding that any change in the dissociation between structure and function after intervention may serve as evidence of efficacy.^14^ Despite the planned and upcoming trials of novel therapies, a lack of longitudinal data, particularly for OCT and fundus autofluorescence (FAF) imaging, is apparent in the literature.^8^^,^^20^, ^21^, ^22^, ^23^, ^24^, ^25^

Herein, we present a retrospective natural history study in a large cohort of adults and children with variants in *GUCY2D*, which provides a detailed description of the genotypic and phenotypic features, with a long duration of follow-up. This information is of particular importance for improving genetic counseling and advice on prognosis, and provides a crucial step toward the design of a therapeutic clinical trial in *GUCY2D*-LCA/EOSRD, as well as identifying a cohort of molecularly confirmed patients who may participate in such future trials.

## Methods

This retrospective study protocol adhered to the tenets of the Declaration of Helsinki and received approval from the Moorfields Eye Hospital ethics committee. Informed consent was obtained from all adult subjects, whereas informed consent and assent were obtained from parents and children, respectively.

### Patient Identification

Patients were identified from the genetic retina clinics at a single tertiary referral center (Moorfields Eye Hospital, London, UK). In total, 22 patients with likely disease-causing variants in *GUCY2D* were ascertained for detailed phenotyping.

### Molecular Diagnosis

Genomic DNA was isolated from peripheral blood lymphocytes (Gentra Puregene Blood Extraction Kit; Qiagen, Venlo, Netherlands). A combination of Sanger sequencing and next-generation sequencing, including a panel of retinal dystrophy genes, whole exome sequencing (WES), and whole genome sequencing, was used to identify variants in *GUCY2D*. All patients with 1 allele identified from WES were subjected to Sanger sequencing of the first coding exon of the gene to check for a second allele, owing to the lack of coverage of the *GUCY2D* first coding exon by WES. Mutation nomenclature was assigned in accordance with GenBank accession number NM_000180.

Minor allele frequency for the identified variants in the general population was assessed in the Genome Aggregation Database (gnomAD) datasets (http://gnomad.broadinstitute.org/; accessed on December 12, 2018) (Supplementary Table 1; Supplemental Material available at AJO.com). Prediction of pathogenicity was assessed using the predictive algorithms of Polymorphism Phenotyping v2 (PolyPhen2, http://genetics.bwh.harvard.edu/pph2/; accessed on December 12, 2018) and Sorting Intolerant from Tolerant (SIFT, http://sift.jcvi.org/; accessed on December 12, 2018) (Supplementary Table 1). Where relevant, disruption of potential splice sites was assessed using Human Splicing Finder (http://www.umd.be/HSF3/; accessed on December 12, 2018) (Supplementary Table 1). Variants likely to affect function were assessed for segregation in available family members.

### Clinical Assessment

All available clinical notes were reviewed. Visual acuity (VA), refraction, funduscopy, and slit-lamp biomicroscopy findings were extracted. All patients were seen by medical retina specialists in the genetics/medical retina clinic. Age of onset is defined as the age at which the family first noticed any symptoms and sought medical care. Age seen is the age at which the patient was first seen at our referral center.

Best-corrected logMAR visual acuity (BCVA) was assessed, monocularly, with an Early Treatment Diabetic Retinopathy Study chart. Patients were read standardized instructions. Precision Vision lightboxes were used (Precision Vision, Woodstock, Illinois, USA) and were illuminated with 2 cool daylight 20 watt fluorescent tubes, with the overhead lights turned off, so that no more than 161.4 lux should fall at the center of the chart. LogMAR values were calculated from the number of letters read, where the higher the logMAR value, the worse the BCVA. Subjective and objective refraction was undertaken by a specialist optometrist for both adults and children.

### Electrophysiological Assessment

Full-field ERG and pattern electroretinography were performed using gold foil corneal electrodes and incorporated the International Society for Clinical Electrophysiology of Vision (ISCEV) standards,^26^^,^^27^ except in infants and young children, who underwent ERG testing with skin electrodes without mydriasis using modified protocols.^28^

### Retinal Imaging

Color fundus imaging was obtained by conventional 35-degree fundus imaging (Topcon Great Britain Ltd, Berkshire, UK) or ultra-widefield (200-degree) confocal scanning laser imaging (Optos plc, Dunfermline, UK). FAF imaging was performed using 30-degree or 55-degree Spectralis (Heidelberg Engineering Ltd, Heidelberg, Germany), or Optos (Optos plc) imaging. Spectral-domain OCT scans (Spectralis; Heidelberg Engineering Ltd) were used to assess cross-sectional and longitudinal structural changes.

## Results

### Demographics

The cohort included 21 patients (female n = 11) from 19 families, with an age range at first examination in our hospital of 0-54 years. The length of follow-up ranged from 1 to 56 years (mean ± standard deviation [SD], 10 ± 11.96 years).

### Molecular Genetics

**Figure 1:**
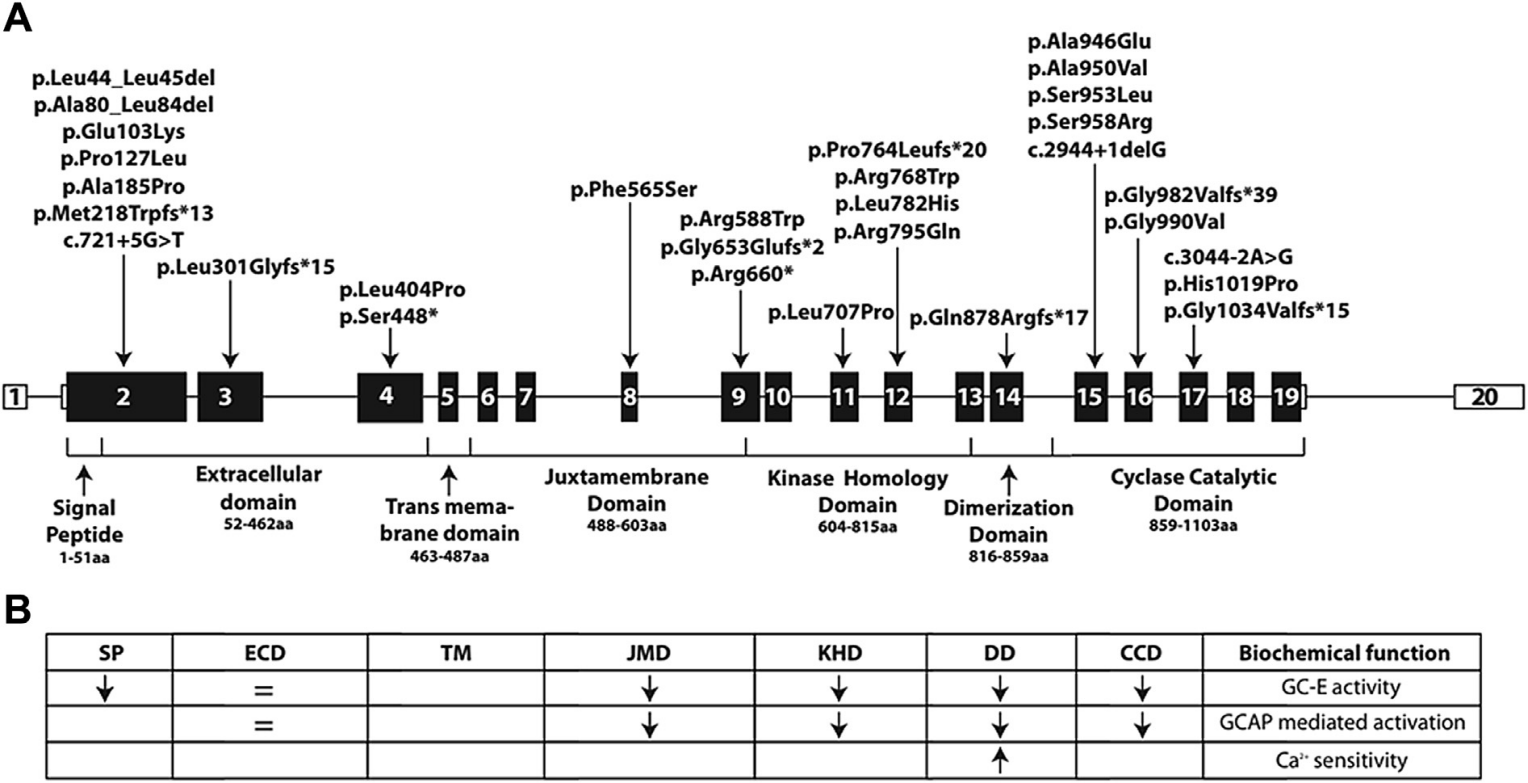
*GUCY2D* gene and protein domains. (A) Schematic diagram showing the *GUCY2D* gene, protein domains, and the location of variants identified in our cohort. (B) Predicted or experimentally determined effect of reported variants on guanylate cyclase function. Variants in the transmembrane domain have not been experimentally investigated. Arrows indicate decrease (↓) and increase (↑) of the function. CCD = cyclase catalytic domain; DD = dimerization domain; ECD = extracellular domain; GC-E = guanylate cyclase 2D; JMD = juxtamembrane domain; KHD = kinase homology domain; SP = signal peptide; TMD = transmembrane domain.

Table 1 and Figure 1 summarize the molecular findings in our cohort. Two pedigrees contributed more than 1 patient (Patients P2A and P2B are siblings, as well as P8A and P8B), with the remaining 17 patients being simplex cases. All patients had 2 likely disease-causing variants in *GUCY2D*. Fourteen patients were compound heterozygotes and 7 patients harbored homozygous variants. The variant minor allele frequencies in the general population (gnomAD database) are reported in Supplementary Table 1. The predicted effect of the variants identified in our cohort is summarized in Table 2. Out of a total of 29 rare variants identified, 14 have not been previously reported in retinal dystrophies. The variants identified in our cohort were scattered throughout the full length of the gene, from exon 2 to exon 17. The majority of the variants (n = 15) are missense, in agreement with previous studies.^7^ Nine small insertions or deletions that cause a frameshift or in-frame deletion and a small number of splice site (n = 3) and premature stop codon (n = 2) variants were identified.

**Table 1:** Variants in the *GUCY2D* Cohort

P	Family ID	Con.	Hom. variant	Variant 1	Protein Effect	Variant Type	PUV	Variant 2	Protein Effect	Variant Type	PUV
P1	GC12356			c.307G>A^†^	p.(Glu103Lys)^†^	Missense		c.238_252delGCCGCCGCCCGCCTG	p.(Ala80_Leu84del)	In-frame deletion	
P2A	GC19319			c.307G>A^†^	p.(Glu103Lys)^†^	Missense		c.1762C>T	p.(Arg 588Trp)	Missense	
P2B	GC19319			c.307G>A^†^	p.(Glu103Lys)^†^	Missense		c.1762C>T	p.(Arg 588Trp)	Missense	
P3	GC1015			c.380C>T	p.(Pro127Leu)	Missense		c.901_908delCTTCGCAG	p.(Leu301Glyfs*15)	Frameshift	
P4	GC17851			c.553G>C	p.(Ala185Pro)	Missense	✓	c.721+5G>T		Splicing	✓
P5	GC19719			c.307G>A^†^	p.(Glu103Lys)^†^	Missense		c.2872A>C	p.(Ser958Arg)	Missense	✓
P6	GC3264		✓	c.652delA	p.(Met218Trpfs*13)	Frameshift	✓				
P7	GC22697			c.2837C>A	p.(Ala946Glu)	Missense	✓	c.2969G>T	p.(Gly990Val)	Missense	✓
P8A	GC19606		✓	c.3056A>C	p.(His1019Pro)	Missense					
P8B	GC19606		✓	c.3056A>C	p.(His1019Pro)	Missense					
P9	GC16211		✓	c.3098_3099insCGTGCTCT	p.(Gly1034Valfs*15)	Frameshift					
P10	GC16935			c.1343C>A	p.(Ser448*)	Nonsense		c.1958delG	p.(Gly653Glufs*2)	Frameshift	✓
P11	GC16929			c.2302C>T	p.(Arg768Trp)	Missense		c.1978C>T	p.(Arg660*)	Nonsense	
P12	GC18677			c.2384G>A	p.(Arg795Gln)	Missense		c.1211T>C	p.(Leu404Pro)	Missense	✓
P13	GC1036			c.307G>A^†^	p.(Glu103Lys)^†^	Missense		c.2849C>T	p.(Ala950Val)	Missense	
P14	GC17418		✓	c.c.2120T>C	p.(Leu707Pro)	Missense	✓				
P15	GC24539	✓	✓	c.3044-2A>G		Splicing	✓				
P16	GC18674			c.2944+1delG		Splicing	✓	c.2858C>T	p.(Ser953Leu)	Missense	✓
P17	GC24284	✓		c.1694T>C	p.(Phe565Ser)	missense		c.2633_2636delAAGT	p.(Gln878Argfs*17)	Frameshift	✓
P18	GC17645		✓	c.129_134delTCTGCT	p.(Leu44_Leu45del)	In-frame deletion					
P19	GC17984			c.2944delG	p.(Gly982Valfs*39)	Frameshift		c.2291delC	p.(Pro764Leufs*20)	Frameshift	✓

P and GC no = patient identifier; Con. = consanguinity; Hom. = Homozygous; PUV = previously unreported variant; † = same variants; A and B denotes siblings

**Table 2:** Predicted Effect of *GUCY2D* Variants

Effect	N^a^	Frequency
Frameshift	9	21.4%
In-frame deletion	3	7.1%
Missense	25	59.5%
Nonsense	1	2.4%
Splicing defect	4	9.5%

a Total n = 42, 2 alleles per patient.

All the missense variants were predicted to be “probably damaging” or “possibly damaging” by PolyPhen2 and “damaging” by SIFT (Supplementary Table 1). The 3 variants at donor or acceptor splice sites were predicted to alter splicing by Human Splice Finder (Supplementary Table 1). The most common variant in our cohort was p.(Glu103Lys), present in 5 patients from 4 different families in the compound heterozygous state.

### Symptoms and Clinical Examination Findings

All patients developed nystagmus and marked reduction of VA within the first 3 years of life, with 15 patients (71%) having documented nystagmus in the first 3 months of life. The primary working hypothesis for all patients was LCA/EOSRD. In 12 patients (57%) photophobia was a prominent symptom, and 8 patients (38%) experienced nyctalopia. Other recorded symptoms included glare in daylight and reduced color vision (n = 5); Patients P3 and P16 failed all Ishihara color test plates, P4 scored 7 of 17 plates, and P2A and P2B reported that they had never been able to appreciate colors.

Other presentations were also documented including lack of eye contact or attention to faces or large toys, as well as significant eye poking. Keratoconus (KC) was observed in 4 patients (19%; P6, P10, P13, and P19, age range of KC presentation 8-40 years, mean ± SD 27.6 ± 13.67 years), with P13 having a right corneal graft (secondary to KC). Three patients developed cataract in childhood (P6, P8B, and P10).

### Visual Acuity

BCVA at first clinic review ranged from 0.4 logMAR to no perception of light, with an age range of 0-54 years. Only 5 patients (24%) were able to record a VA on a Snellen chart, with patient P4 having the best BCVA at 0.40 logMAR in the right eye, 0.54 in the left eye at 11 years of age. P3 and P5 had VA of 0.48 and 0.78 logMAR in their better-seeing eyes at the ages of 8 and 4 years, respectively. The remaining 2 of the 5 patients had VA at 1.2 and 1.5 logMAR in their better-seeing eye, initially measured within their fourth decade of life. Seventy-six percent of our cohort (n = 16) were severely visually impaired, with BCVA of hand movements or worse. BCVA is summarized in Table 3 and presented in detail in Supplementary Table 2 (Supplemental Material available at AJO.com).

**Table 3:** Refraction and Visual Acuity in the *GUCY2D* Cohort

	N	%
VA in best-seeing eye, at initial examination:		
NPL	4	19%
PL	4	19%
HM	4	19%
Fixate on large objects	3	14%
0.4 logMAR	1	4%
0.48 logMAR	1	4%
0.78 logMAR	1	4%
1.2 logMAR	1	4%
1.5 logMAR	1	4%
1.56 logMAR	1	4%
Refraction:		
Hyperopia	12	57%
Myopia	3	14%
Plano	2	9%
Not available	4	19%

HM = hand motions; NPL = no perception of light; PL = perception of light; VA = visual acuity.

All patients reported a subjective stability over time. However, Patient P3 noted some deterioration in central vision, recorded as changing from 0.78 logMAR in each eye at initial presentation at the age of 4 years to 1.0 logMAR and 1.3 logMAR for the right and left eye, respectively, at the age of 47 years. Patient P4, who presented with the best VA, maintained a stable VA of 0.48 logMAR and 0.6 logMAR for right and left eyes, respectively, until her latest follow-up at age 23 years.

### Refraction

Seventeen patients had refraction data available, with 12 (57%) being hyperopic, of whom 50%have a refractive error of greater than +6.5 diopters (D). Myopia was observed only in 3 patients (14%), with P18 being highly myopic (OD: –7.00 D, OS: –8.00 D), and 2 patients did not have a significant refractive error. Refractive error is summarized in Table 3 and presented in more detail in Supplementary Table 2.

### Fundus Findings

**Figure 2:**
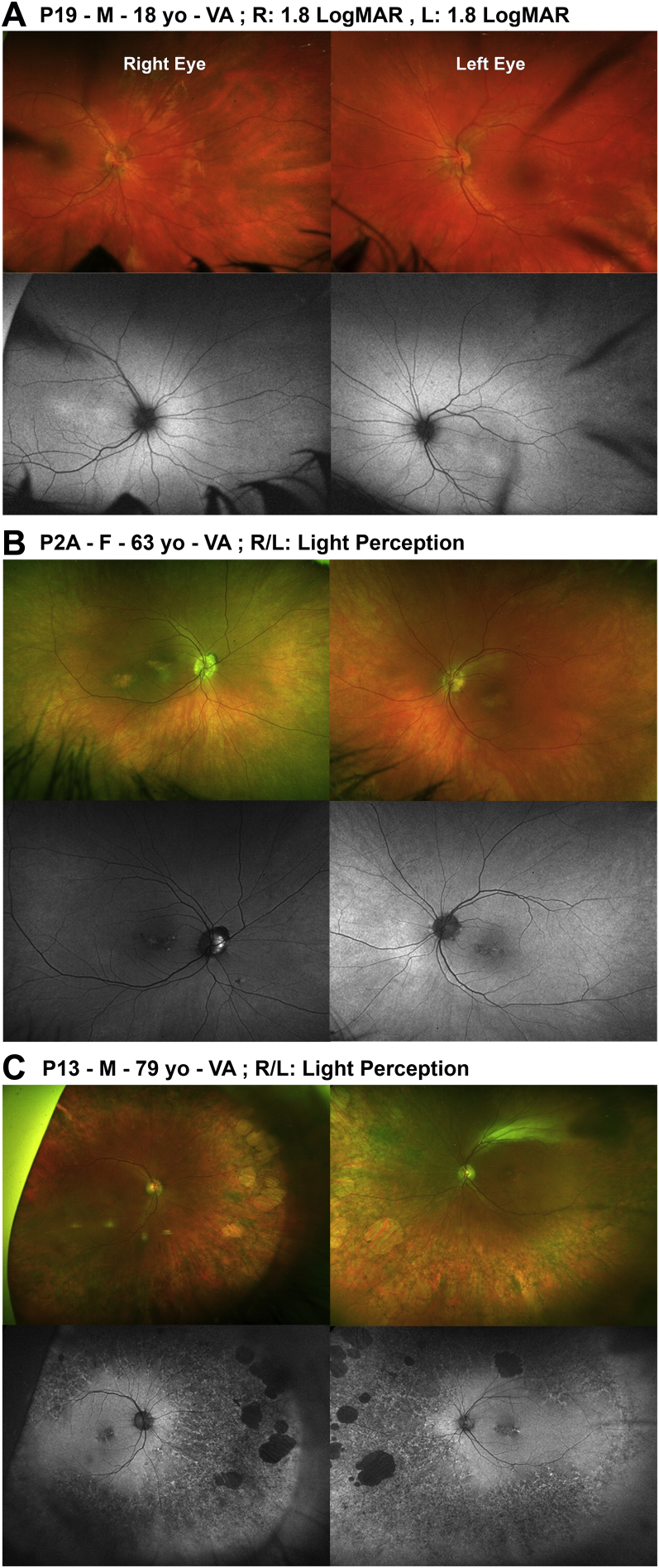
Color fundus photographs and fundus autofluorescence (FAF) imaging of the right and left eyes of 3 patients (Optos plc, Dunfermline, UK). (A) Patient P19. Normal fundus appearance and central foveal hyperautofluorescence on FAF, without mid-peripheral or peripheral changes. (B) Patient P2A. Mild yellow macular atrophy, as well as fine peripheral pigmentary changes. Normal FAF images apart from central small areas of hypoautofluorescence. (C) Patient P13. Extensive peripheral retinal pigment epithelium atrophy and pigment hypertrophy with large lacunae of chorioretinal atrophy in far periphery in both eyes. On FAF there is central early hypoautofluorescence with small central foci of hyperautofluorescence and mid- to far-peripheral generalized hypoautofluorescence with granular hyperautofluorescence and discrete scattered large patches of hypoautofluorescence. L = left eye; R = right eye; VA; visual acuity; yo = years old.

On fundus examination, 67% of patients had either normal (n = 11, Figure 2A) or blond fundus appearance (n = 3); (age range 1-27 years at examination; mean ± SD, 14.5 ± 9.3 years). Four patients (19%) had a normal fundus with disc pallor and/or attenuated vessels (age range 1-34 years, mean ± SD, 14.75 ± 16.07 years). Patient P5 had only fine peripheral pigmentary changes. Among the oldest patients, 3 (P2A, P2B [Figure 2B], P3; 14%) had mild yellow macular atrophy, as well as fine peripheral pigmentary changes, examined at age 53, 54, and 43 years, respectively.

Longitudinal data were available for 17 patients (81%) with documented slit-lamp examination findings and/or color fundus imaging. Among those presenting with a normal fundus examination (n = 9), apart from 1 patient (P14), fundus findings remained unchanged until the latest visit, over a follow-up period ranging between 5 and 15 years. Patient P14 developed pale optic discs, attenuated vessels, and fine pigmentary changes in the periphery, with prominent choroidal vasculature at the age of 14 years. Patient P13 had extensive peripheral retinal pigment epithelium (RPE) atrophy and pigment hypertrophy, with large (2-5 disc diameter) lacunae of chorioretinal atrophy in the far periphery in both eyes at 60 years of age. A previous normal fundus examination was documented at the age of 30 years (Figure 2C).

### Electrophysiological Assessment

**Figure 3:**
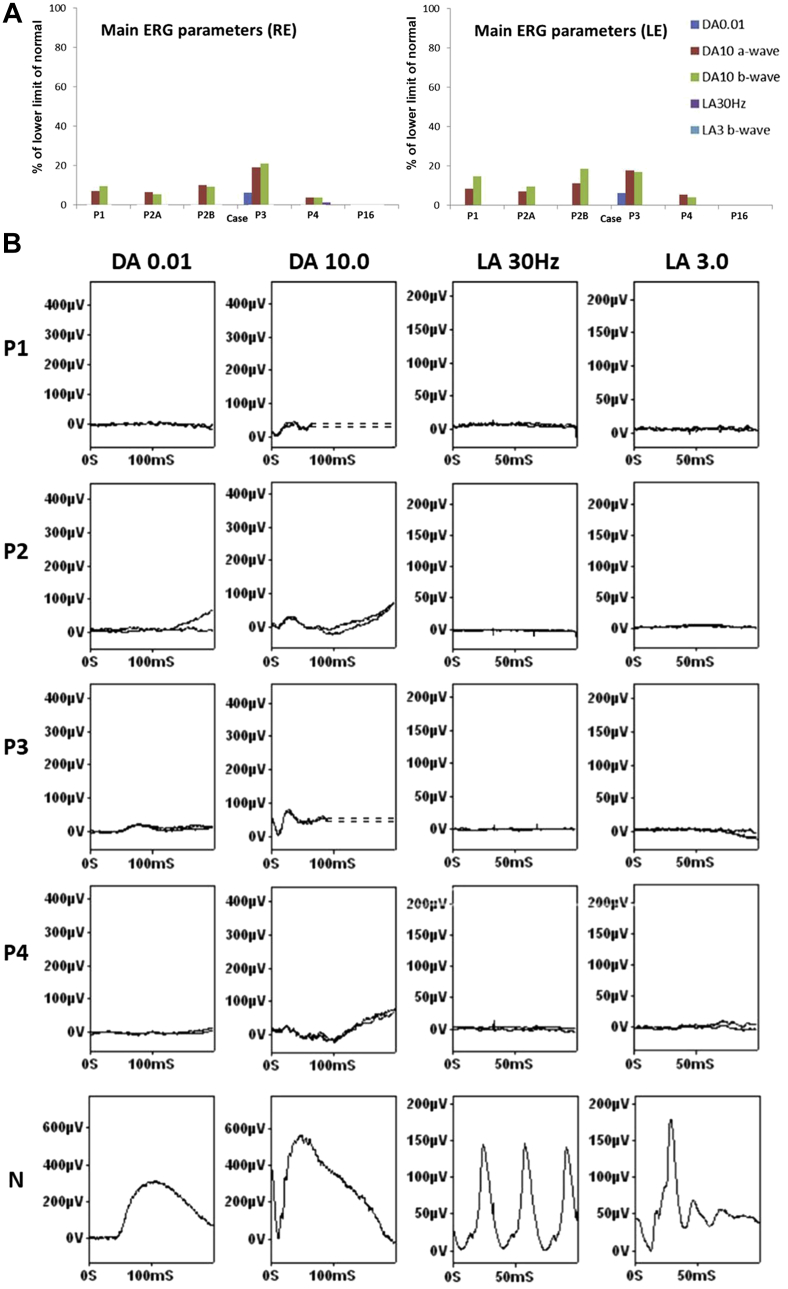
Graphical representation of full-field electroretinography (ERG) findings and examples of recordings. (A) ISCEV-standard full-field ERG amplitudes are plotted as a percentage of the lower limit of normal obtained in a control group for right (RE) and left (LE) eyes. The findings were consistent with a severe cone-rod dystrophy (Patients P1, P2A, P2B, P3, and P4) or severe photoreceptor dystrophy (P16; undetectable ERGs). (B) Examples of ISCEV-standard full-field ERG traces recorded from Patients P1, P2, P3, and P4 and a representative unaffected control subject (N) for comparison. Dark-adapted (DA) ERGs are shown for flash strengths of 0.01 and 10.0 cd.s/m^2^ (DA 0.01; DA 10.0). Light-adapted (LA) ERGs are shown for a flash strength of 3.0 cd.s/m^2^ (LA 3.0; 30 Hz and 2 Hz). Recordings are shown from 1 eye with traces superimposed to demonstrate reproducibility. Note the higher scaling factor used to illustrate low-amplitude DA ERGs compared with the control. Broken lines replace blink artefacts that occur after the ERG b-waves. Pattern ERGs were not recordable owing to the effects of marked nystagmus in all patients.

ISCEV-standard electrophysiological data were available for 6 subjects. The results of full-field ERG testing are summarized in Figure 3A and B. In older children and adults (age range 14-57 years) dark-adapted (DA) dim flash ERGs were undetectable in 5 patients and severely subnormal in 1 patient (P3; 44 years). The strong flash (DA10) ERG a- and b-waves were reduced by approximately 80%-95% in those with a detectable response; b-waves were severely reduced and of abnormally short peak time (24-37 ms) in all 5 cases (P1, P2A, P2B, P3, and P4). Light-adapted (LA) ERGs were undetectable in all but 1 case, with a residual LA 30 Hz flicker ERG in 1 eye (P4). Pattern ERGs were not recordable owing to the effects of marked nystagmus in all. The ERGs were stable in the 2 patients that were monitored over 17 years (P3; first tested age 44 years) and 6 years (P4; first tested age 14 years).

In the 4 infants (aged 5-14 months) and 3 children (aged 6-9 years; P5, P16, and P18) tested with skin electrodes, the flash ERGs were undetectable under DA and LA conditions (P15, P16, P18) or showed only residual responses (P5, P8B, P9, P14), with only DA ERGs being detectable in 2 (P9, P14).

### Optical Coherence Tomography Findings

**Figure 4:**
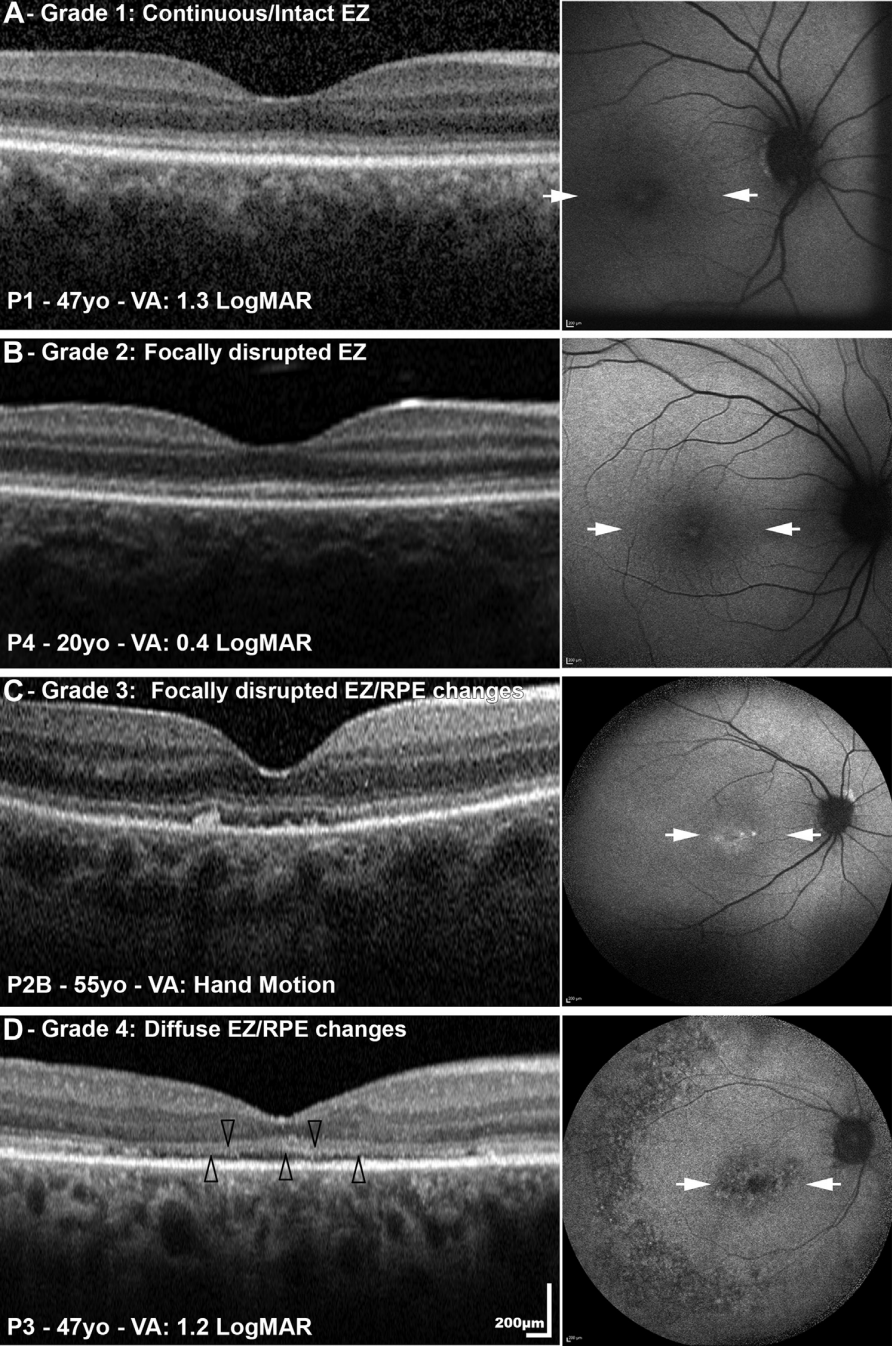
Optical coherence tomography (OCT) and fundus autofluorescence (FAF) imaging of 4 patients (P1, P4, P2B, P3). OCT findings at baseline were grouped into 4 different grades based on ellipsoid zone (EZ) integrity and retinal pigment epithelium (RPE) changes: (A) grade 1: continuous/intact EZ; (B) grade 2: focally disrupted EZ; (C) grade 3: focally disrupted EZ with RPE changes; and (D) grade 4: diffuse EZ and RPE changes. The arrowheads point to the attenuated EZ. The right column presents the corresponding FAF images: (A) normal FAF appearance; (B) central early hypoautofluorescence with small central foci of hyperautofluorescence; (C) normal FAF images apart from central small areas of hypoautofluorescence; and (D) central and mid-peripheral hypoautofluorescence. The white arrows mark the border of the corresponding OCT line scans. VA = visual acuity; yo = years old.

OCT imaging was available for 11 patients. Age at baseline ranged from 7.3 to 76.3 years (mean ± SD, 34.1 ± 22.7 years). OCT findings at baseline were grouped into 4 different grades based on ellipsoid zone (EZ) integrity and RPE changes: (1) continuous/intact EZ (n = 6), (2) focally disrupted EZ (n = 2), (3) focally disrupted EZ with RPE changes (n = 2), and (4) severely disrupted EZ with RPE changes (n = 1). In Figure 4 all 4 grades are represented, at different ages and visual acuities. In Table 4 the OCT data for all patients are summarized. The EZ was present in all patients with available imaging. Longitudinal OCT data were available for 9 patients over a follow-up period between 2.0 and 13.3 years (mean, 5.2 years), without any evidence of progression over time.

**Table 4:** Optical Coherence Tomography Findings in the *GUCY2D* Cohort

Patient	VA (OD/OS)	Age at Baseline OCT (Y)	Follow-up Time (Y)	OCT EZ Appearance at Both Baseline and Follow-up
P1	1.3/1.2	47.3	6.2	Continuous/intact EZ
P2A	1.8/1.5	59.6	4.2	Focally disrupted with RPE changes
P2B	HM	54.8	4.2	Focally disrupted with RPE changes
P3	1.3/1	47.4	13.3	Diffuse EZ/RPE change
P4	0.48/0.6	20.3	3.0	Focally disrupted EZ
P5	0.48/0.62	8.0	4.0	Continuous/intact EZ
P8A	HM	25.4	NA	Continuous/intact EZ^a^
P9	HM	11.3	3.0	Continuous/intact EZ
P13	HM	76.4	2.0	Focally disrupted EZ
P14	PL	7.3	6.4	Continuous/intact EZ
P19	PL	18.2	NA	Continuous/intact EZ^a^

EZ = ellipsoid zone; HM = hand motions; OCT = optical coherence tomography; PL = perception of light; RPE = retinal pigment epithelium; VA = visual acuity.

a No available follow-up OCT scan.

OCT was not available for the remaining 10 patients (age range, 1-27 years), either owing to severe nystagmus or KC or because of young age at last follow-up visit (4 patients younger than 10 years of age). Those with no OCT images had VA of hand movements or worse and normal or blond fundus appearance (n = 8), with no fundus view owing to severe KC in 2 patients (both aged 17 at first examination). Those subjects with OCT available (n = 11) had a similar age range (7.3-76.3) and VA range (0.48 to light perception).

### Fundus Autofluorescence Imaging

FAF images were available for 11 (52%) patients (same patients as had OCT). Patients with an intact EZ had either (1) normal FAF appearance (n = 1, P1 between 42 and 54 years of age, Figure 4A), (2) central foveal hyperautofluorescence (n = 2; P14 between 7 and 14 years of age; P19 at age 18 years, Figure 2A), or (3) a perimacular ring of increased AF (n = 2; P5 between 8 and 12 years of age; P8A at age 25). No abnormal mid-peripheral or peripheral changes were identified in these patients throughout the follow-up period of up to 12 years. Two patients (P2A, Figure 2B, and P2B, Figure 4C) had normal FAF images apart from central small areas of hypoautofluorescence in their 50s and 60s; on OCT they had focally disrupted EZ at the macula with RPE changes (Figure 4C). Patient P3, who had more severely disrupted EZ on OCT, showed central and mid-peripheral hypoautofluorescence on FAF imaging (Figure 4D). Those with focally disrupted EZ with RPE changes (P4, Figure 4B, and P13, Figure 2C) had central early hypoautofluorescence with small central foci of hyperautofluorescence, with the latter also having mid to far peripheral generalized hypoautofluorescence with granular hyperautofluorescence and discrete scattered large patches of hypoautofluorescence. FAF imaging was not available in the remaining patients for the same aforementioned reasons as per OCT imaging, with photoaversion being an additional reason challenging image acquisition.

## Discussion

We describe the phenotype and natural history of a large cohort of patients of a wide range of ages with *GUCY2D-*LCA/EOSRD ascertained at a single UK referral center. The clinical presentation in our cohort is in keeping with previous reports, with early-onset disease, severe visual impairment, and a structure-function dissociation.^15^^,^^20^, ^21^, ^22^, ^23^, ^24^, ^25^^,^^29^, ^30^, ^31^, ^32^ All patients presented with nystagmus and profound visual loss within the first 3 years of life. Long-term follow-up showed stability of VA over time. Keratoconus and early-onset cataract contributed to further loss of VA in a minority of subjects.

Recently Stunkel and associates expanded the retinal disease spectrum associated with autosomal recessive mutations in *GUCY2D*, reporting 5 patients with “congenital night blindness” and evidence of progression to mild retinitis pigmentosa.^33^ We did not identify any similar patients in our cohort with autosomal recessive *GUCY2D*. However, 2 patients (P4 and P5) had marked rod-related symptoms, without macular changes and with some residual cone ERG activity, with stability over 7 years of follow-up in P4. BCVA was better than the rest of the cohort (however, it was significantly worse than the VA in the aforementioned subjects with “congenital night blindness”) and was maintained over the follow-up period. A ring of hyperautofluorescence in the outer macula was observed in 1 of the 2 patients, a common finding in retinitis pigmentosa.^34^, ^35^, ^36^ For the first 3 patients (P1, P2A, and P2B) during their early clinic visits the differential diagnosis included achromatopsia, since their residual visual function was better than might be expected for LCA. If we group the first 6 subjects (P1-P6), who arguably have better-preserved VA, together and compare them to the rest of the cohort, we can explore this further. As presented in Table 1 and in Figure 1A, all 6 subjects had 1 disease-causing missense variant in exon 2 encoding the extracellular domain, and it appears that variants in the extracellular domain do not alter the biochemical activity of GC-E.^10^, ^11^, ^12^ Patients harboring variants in exon 2 seem to have a milder phenotype, characterized by better visual acuity, which is preserved over time. However, these patients still had severe generalized impairment of retinal function on ERG testing.

International standard full-field ERGs showed evidence of a severe cone-rod dystrophy in 5 of 6 patients and undetectable ERGs in 1 other, in keeping with a severe photoreceptor dystrophy. Detectable but subnormal DA 10 ERG b-waves were of unusually short peak time; the absence of detectable LA ERGs in 9 of 10 eyes and presence of DA ERGs to a dim flash (below cone system threshold) in subject P3 suggests a rod-mediated origin, although the mechanism is uncertain. The ERGs in younger children and infants were consistent with severe cone-rod or severe photoreceptor dystrophy. The similarity of ERG phenotypes across a wide range of ages and the lack of ERG deterioration in serial recordings from 2 subjects suggests severe early-onset disease but with relative stability or slow progression with age. Similar stability was also observed with FAF and OCT imaging, and in addition in the retinal appearance on funduscopy. The imaging findings were not correlated with visual function; namely, despite having profoundly reduced VA and severely abnormal full-field ERGs, the EZ was present in the majority of patients.

These OCT findings differ from those found in the majority of other forms of LCA, where patients have extensive photoreceptor cell loss.^13^ OCT in *GUCY2D*-LCA/EOSRD has only been reported in a few studies and these were consistent with our findings.^14^^,^^15^^,^^20^ A retrospective study included 3 patients aged between 20 and 53 years, with unremarkable retinal lamination, described as less well-defined than normal.^13^ OCT imaging in another cohort of 11 patients, ranging in age from 6 months to 37 years, showed that all patients had intact rod photoreceptors but abnormalities in foveal cones.^15^ A recent study (n = 28 subjects, aged 2-59 years) reported a dissociation between structure and function, as revealed by retinal layer abnormalities on OCT and full-field sensitivity testing.^14^ In the same study, Jacobson and associates identified outer nuclear layer thinning over the fovea and decreased intensity of the EZ reflectivity.^14^ Further evaluation of retinal structure with adaptive optics ophthalmoscopy may be of value,^37^ in order to further elucidate the photoreceptor structure in these patients at a cellular level; however, this will likely be challenging in many patients owing to poor fixation/nystagmus, keratoconus, and early-onset cataract.

The aforementioned disconnect between structure and function raises the potential for functional rescue and possible amenability to gene-based therapy. A successful therapeutic approach has been examined in the GUCY1*B chicken model.^38^ In this study, a lentivirus vector delivering bovine *GUCY2D* was injected into chicken embryos. Six of the 7 treated embryos exhibited improvement in VA and ERG responses. Moreover, the retinal degeneration was slower in comparison to the untreated chickens. However, they reported that disease development was not preventable despite delivering gene replacement at an early stage.^7^^,^^38^ In another study, 3-week-old knockout mice were injected with subretinal AAV-*GUCY2D* (bovine). Although successful restoration of cone arrestin translocation was achieved 5 weeks after the injection, there was no restoration of cone ERG responses.^7^^,^^17^ However, a study delivering subretinal AAV5 containing human *GUCY2D* to the knockout mouse model showed not only an efficient transgene expression in rod and cone photoreceptors, but also successful restoration of cone function, as well as the activity of the GC enzyme. Moreover, this restoration of retinal function persisted for at least 6 months. Similar results up to 6 months post injection were observed in treated *Gucy2e*^-/-^ mice with rAAV2/8 vector: dose-dependent restoration of rod and cone function and an improvement in visual behavior.^18^ These promising studies have raised the likelihood of gene-replacement trials for patients with *GUCY2D*-LCA/EOSRD.^7^^,^^18^^,^^39^ Determining the outcome measures, characterization of large cohorts of potential participants, and defining disease natural history are fundamental steps toward the optimal design of these gene therapy trials.

Our study has provided valuable information about the clinical phenotype and natural history of *GUCY2D*-LCA/EOSRD, established a well-characterized cohort of molecularly confirmed potential trial participants, and reported potential genotype-phenotype correlations. It has, in addition, highlighted the relative structural and functional stability over a broad age range, thereby indicating a wide therapeutic window to be exploited by planned and anticipated interventional trials.

## Supplemental Data

Data Profile

## References

[1] Chung D.C., Traboulsi E.I. (2009). Leber congenital amaurosis: clinical correlations with genotypes, gene therapy trials update, and future directions. J AAPOS.

[2] Kumaran N., Moore A.T., Weleber R.G., Michaelides M. (2017). Leber congenital amaurosis/early-onset severe retinal dystrophy: clinical features, molecular genetics and therapeutic interventions. Br J Ophthalmol.

[3] Stone E.M. (2007). Leber congenital amaurosis - a model for efficient genetic testing of heterogeneous disorders: LXIV Edward Jackson Memorial Lecture. Am J Ophthalmol.

[4] Kumaran N., Pennesi M., Yang P., Adam M.P., Ardinger H., Pagon R.A. (2018). Leber congenital amaurosis/early-onset severe retinal dystrophy overview. GeneReviews® [Internet].

[5] Francis P.J. (2006). Genetics of inherited retinal disease. J R Soc Med.

[6] Daiger S.P., Sullivan L.S., Bowne S.J. (2018). Retinal Information Network.

[7] Sharon D., Wimberg H., Kinarty Y., Koch K.W. (2018). Genotype-functional-phenotype correlations in photoreceptor guanylate cyclase (GC-E) encoded by GUCY2D. Prog Retin Eye Res.

[8] Perrault I., Rozet J.M., Gerber S. (2000). Spectrum of retGC1 mutations in Leber's congenital amaurosis. Eur J Hum Genet.

[9] Gill J.S., Georgiou M., Kalitzeos A. (2019). Progressive cone and cone-rod dystrophies: clinical features, molecular genetics and prospects for therapy. Br J Ophthalmol.

[10] Duda T., Pertzev A., Sharma R.K. (2011). 657WTAPELL663 motif of the photoreceptor ROS-GC1: a general phototransduction switch. Biochem Biophys Res Commun.

[11] Duda T., Venkataraman V., Goraczniak R. (1999). Functional consequences of a rod outer segment membrane guanylate cyclase (ROS-GC1) gene mutation linked with Leber's congenital amaurosis. Biochemistry.

[12] Rozet J.M., Perrault I., Gerber S. (2001). Complete abolition of the retinal-specific guanylyl cyclase (retGC-1) catalytic ability consistently leads to leber congenital amaurosis (LCA). Invest Ophthalmol Vis Sci.

[13] Pasadhika S., Fishman G.A., Stone E.M. (2010). Differential macular morphology in patients with RPE65-, CEP290-, GUCY2D-, and AIPL1-related Leber congenital amaurosis. Invest Ophthalmol Vis Sci.

[14] Jacobson S.G., Cideciyan A.V., Sumaroka A. (2017). Defining outcomes for clinical trials of Leber congenital amaurosis caused by GUCY2D mutations. Am J Ophthalmol.

[15] Jacobson S.G., Cideciyan A.V., Peshenko I.V. (2013). Determining consequences of retinal membrane guanylyl cyclase (RetGC1) deficiency in human Leber congenital amaurosis en route to therapy: residual cone-photoreceptor vision correlates with biochemical properties of the mutants. Hum Mol Genet.

[16] Zhang T., Jiang W., Song X., Zhang D. (2016). The association between visual impairment and the risk of mortality: a meta-analysis of prospective studies. J Epidemiol Commun Health.

[17] Haire S.E., Pang J., Boye S.L. (2006). Light-driven cone arrestin translocation in cones of postnatal guanylate cyclase-1 knockout mouse retina treated with AAV-GC1. Invest Ophthalmol Vis Sci.

[18] Mihelec M., Pearson R.A., Robbie S.J. (2011). Long-term preservation of cones and improvement in visual function following gene therapy in a mouse model of leber congenital amaurosis caused by guanylate cyclase-1 deficiency. Hum Gene Ther.

[19] Aguirre G.K., Butt O.H., Datta R. (2017). Postretinal structure and function in severe congenital photoreceptor blindness caused by mutations in the GUCY2D gene. Invest Ophthalmol Vis Sci.

[20] Simonelli F., Ziviello C., Testa F. (2007). Clinical and molecular genetics of Leber's congenital amaurosis: a multicenter study of Italian patients. Invest Ophthalmol Vis Sci.

[21] Wang S., Zhang Q., Zhang X. (2016). Clinical and genetic characteristics of Leber congenital amaurosis with novel mutations in known genes based on a Chinese eastern coast Han population. Graefes Arch Clin Exp Ophthalmol.

[22] Walia S., Fishman G.A., Jacobson S.G. (2010). Visual acuity in patients with Leber's congenital amaurosis and early childhood-onset retinitis pigmentosa. Ophthalmology.

[23] Srilekha S., Arokiasamy T., Srikrupa N.N. (2015). Homozygosity mapping in Leber congenital amaurosis and autosomal recessive retinitis pigmentosa in South Indian families. PLoS One.

[24] Hosono K., Harada Y., Kurata K. (2015). Novel GUCY2D gene mutations in Japanese male twins with Leber congenital amaurosis. J Ophthalmol.

[25] Gradstein L., Zolotushko J., Sergeev Y.V. (2016). Novel GUCY2D mutation causes phenotypic variability of Leber congenital amaurosis in a large kindred. BMC Med Genet.

[26] McCulloch D.L., Marmor M.F., Brigell M.G. (2015). ISCEV Standard for full-field clinical electroretinography (2015 update). Doc Ophthalmol.

[27] Bach M., Brigell M.G., Hawlina M. (2013). ISCEV standard for clinical pattern electroretinography (PERG): 2012 update. Doc Ophthalmol.

[28] Holder G.E., Robson A.G. (2006). Paediatric electrophysiology: a practical approach. Essentials in Ophthalmology.

[29] Dharmaraj S., Silva E., Pina A.L. (2000). Mutational analysis and clinical correlation in Leber congenital amaurosis. Ophthalmic Genet.

[30] Lotery A.J., Namperumalsamy P.P., Jacobson S.G. (2000). Mutation analysis of 3 genes in patients with leber congenital amaurosis. Arch Ophthalmol.

[31] Perrault I., Hanein S., Gerber S. (2005). A novel mutation in the GUCY2D gene responsible for an early onset severe RP different from the usual GUCY2D-LCA phenotype. Hum Mutat.

[32] Yzer S., Leroy B.P., De Baere E. (2006). Microarray-based mutation detection and phenotypic characterization of patients with Leber congenital amaurosis. Invest Ophthalmol Vis Sci.

[33] Stunkel M.L., Brodie S.E., Cideciyan A.V. (2018). Expanded retinal disease spectrum associated with autosomal recessive mutations in GUCY2D. Am J Ophthalmol.

[34] Tee J.J.L., Kalitzeos A., Webster A.R. (2018). Quantitative analysis of hyperautofluorescent rings to characterize the natural history and progression in RPGR-associated retinopathy. Retina.

[35] Murakami T., Akimoto M., Ooto S. (2008). Association between abnormal autofluorescence and photoreceptor disorganization in retinitis pigmentosa. Am J Ophthalmol.

[36] Robson A.G., El-Amir A., Bailey C. (2003). Pattern ERG correlates of abnormal fundus autofluorescence in patients with retinitis pigmentosa and normal visual acuity. Invest Ophthalmol Vis Sci.

[37] Georgiou M., Kalitzeos A., Patterson E.J. (2018). Adaptive optics imaging of inherited retinal diseases. Br J Ophthalmol.

[38] Williams M.L., Coleman J.E., Haire S.E. (2006). Lentiviral expression of retinal guanylate cyclase-1 (RetGC1) restores vision in an avian model of childhood blindness. PLoS Med.

[39] Boye S.E., Alexander J.J., Boye S.L. (2012). The human rhodopsin kinase promoter in an AAV5 vector confers rod- and cone-specific expression in the primate retina. Hum Gene Ther.

