# Supplementary material for: GUCY2D-Associated Leber Congenital Amaurosis: A Retrospective Natural History Study in Preparation for Trials of Novel Therapies
Source: Am J Ophthalmol. 2020 Feb;210:59–70. doi: 10.1016/j.ajo.2019.10.019 (PMC7013380; doi:10.1016/j.ajo.2019.10.019)
Supplement: Supplementary Table 1 [file mmc2.pdf]

Supplementary table 1. *GUCY2D* variants characterisation

Patient	Family ID	Variant	Protein effect	Variant type	gnomAD MAF	Polyphen2 prediction	SIFT prediction	Human Splicing Finder prediction
P1	GC12356	c.307G>A*	p.(Glu103Lys)*	missense	N/A	probably damaging (1)	Damaging (0.049)	
		c.238_252delGCCGCCGCCCGCCTG	p.(Ala80_Leu84del)	inframe deletion	0.00002134			
P2A	GC19319	c.307G>A*	p.(Glu103Lys)*	missense	N/A	probably damaging (1)	Damaging (0.049)	
		c.1762C>T	p.(Arg588Trp)	missense	N/A	probably damaging (1)	Damaging (0)	
P2B	GC19319	c.307G>A*	p.(Glu103Lys)*	missense	N/A	probably damaging (1)	Damaging (0.049)	
		c.1762C>T	p.(Arg588Trp)	missense	N/A	probably damaging (1)	Damaging (0)	
P3	GC1015	c.380C>T	p.(Pro127Leu)	missense	0.00002115	probably damaging (0.999)	Damaging (0.007)	
		c.901_908delCTTCGCAG	p.(Leu301Glyfs*15)	frameshift	N/A			
P4	GC17851	c.553G>C	p.(Ala185Pro)	missense	N/A	probably damaging (1)	Damaging (0.009)	
		c.721+5G>T		splicing	0.00000438			Alteration of the WT donor site, most probably affecting splicing.
P5	GC19719	c.307G>A*	p.(Glu103Lys)*	missense	N/A	probably damaging (1)	Damaging (0.049)	
		c.2872A>C	p.(Ser958Arg)	missense	N/A	probably damaging (0.975)	Damaging (0.028)	
P6	GC3264	c.652delA	p.(Met218Trpfs*13)	frameshift	N/A			
P7	GC22697	c.2837C>A	p.(Ala946Glu)	missense	N/A	probably damaging (1)	Damaging (0.001)	
		c.2969G>T	p.(Gly990Val)	missense	N/A	probably damaging (1)	Damaging (0)	
P8A	GC19606	c.3056A>C	p.(His1019Pro)	missense	0.00000408	probably damaging (1)	Damaging (0.003)	
P8B	GC19606	c.3056A>C	p.(His1019Pro)	missense	0.00000408	probably damaging (1)	Damaging (0.003)	
P9	GC16211	c.3098_3099insCGTGCTCT	p.(Gly1034Valfs*15)	frameshift	N/A			
P10	GC16935	c.1343C>A	p.(Ser448*)	nonsense	0.00003313			
		c.1958delG	p.(Gly653Glufs*2)	frameshift	N/A			
P11	GC16929	c.2302C>T	p.(Arg768Trp)	missense	0.00014149	probably damaging (1)	Damaging (0)	
		c.1978C>T	p.(Arg660*)	nonsense	0.00000398			
P12	GC18677	c.2384G>A	p.(Arg795Gln)	missense	N/A	probably damaging (1)	Damaging (0)	
		c.1211T>C	p.(Leu404Pro)	missense	N/A	probably damaging (0.988)	Damaging (0.001)	
P13	GC1036	c.307G>A*	p.(Glu103Lys)*	missense	N/A	probably damaging (1)	Damaging (0.049)	
		c.2849C>T	p.(Ala950Val)	missense	N/A	probably damaging (0.976)	Damaging (0.034)	
P14	GC17418	c.c.2120T>C	p.(Leu707Pro)	missense	N/A	probably damaging (1)	Damaging (0.001)	
P15	GC24539	c.3044-2A>G		splicing	N/A			Alteration of the WT aceptor site, most probably affecting splicing.
P16	GC18674	c.2944+1delG		splicing	0.000467209			Alteration of the WT donor site, most probably affecting splicing.
		c.2858C>T	p.(Ser953Leu)	missense	N/A	possibly damaging (0.675)	Damaging (0.005)	
P17	GC24284	c.1694T>C	p.(Phe565Ser)	missense	0.00000398	probably damaging (0.998)	Damaging (0.022)	
		c.2633_2636delAAGT	p.(Gln878Argfs*17)	frameshift	N/A			
P18	GC17645	c.129_134delTCTGCT	p.(Leu44_Leu45del)	inframe deletion	0.00168588			
P19	GC17984	c.2944delG	p.(Gly982Valfs*39)	frameshift	N/A			
		c.2291delC	p.(Pro764Leufs*20)	frameshift	0.00000398			
