# Supplementary material for: GUCY2D-Associated Leber Congenital Amaurosis: A Retrospective Natural History Study in Preparation for Trials of Novel Therapies
Source: Am J Ophthalmol. 2020 Feb;210:59–70. doi: 10.1016/j.ajo.2019.10.019 (PMC7013380; doi:10.1016/j.ajo.2019.10.019)
Supplement: Supplementary Table 2 [file mmc3.pdf]

Supplementary table 2: Clinical Data

Subject	Age of onset	Principal Symptom(s)*	Age (y)	Fundus Appearance	VA (LogMAR)	Latest refractive error
**P1**	< 3mo	Nystagmus	34	Unremarkable<br>Disc pallor<br>Thin vessels	R: 1.3<br>L: 1.2	R: +1.00/-2.00 x 70°<br>L: +1.00
			56	Unchanged	R: 1.3<br>L: 1.3	
**P2A**	1-3yo	Nystagmus<br>Photophobia	53	Poorly defined macula,<br>Central RPE atrophy.<br>Hyperpigmented area inferotemporal to the right macula	R: 1.8<br>L: 1.5	R: +1.50/-0.50 x 20°<br>L: +1.75/-1.75 x 160°
			63	Unchanged	R: 1.8<br>L: 1.6	
**P2B**	3-12mo	Nystagmus<br>Photophobia	54	Poorly defined macula<br>RPE atrophy<br>Small foci of hypertrophy	BE: HM	Emmetropia
			58	Unchanged	BE: HM	
**P3**	< 3mo	Nystagmus<br>Photophobia	4	Central and mid peripheral RPE atrophy	BE: 0.78	R: -0.50/-4.00 x 10°<br>L: +0.50/-4.00 x 170°
			60	Unchanged	R: 1.3<br>L: 1.0	
**P4**	< 3mo	Nyctalopia<br>Reduced color vision	14	Blonde fundus	R: 0.4<br>L: 0.5	R: -3.75/-1.00 x 5°<br>L: -3.75/-2.50 x 180°
			23	Unchanged	R: 0.48<br>L: 0.6	
**P5**	< 3mo	Nystagmus<br>No eye contact<br>Nyctalopia<br>Glare in daytime	8	Peripheral hypopigmentation	R: 0.48<br>L: 0.62	R: +2.00/-2.25 x 5°<br>L: +2.50/-2.25 x 180°
			11	Unchanged	R: 0.48<br>L: 0.54	
**P6**	< 3mo	Nystagmus<br>Eye poking<br>Nyctalopia	17	Unremarkable	BE: NPL	NA
			27	Unchanged	BE: NPL	
**P7**	< 3mo	Nystagmus<br>Eye poking<br>Nyctalopia	1	Blonde fundus	BE: NPL	BE: +7.00
			2	Unchanged	BE: NPL	
**P8A**	< 3mo	Nystagmus<br>Nyctalopia<br>Eye poking	14	Unremarkable fundus apart from thin vessels	BE: HM	NA
			12	Unchanged	BE: HM	
**P8B**	< 3mo	Nystagmus<br>Not fixing<br>Eye poking	1	Unremarkable	BE: fixate at large object	BE: +7.00
			11	Unchanged	BE: HM	
**P9**	3-12mo	Nystagmus<br>Photophobia<br>Nyctalopia	1	Unremarkable	BE: fixate at large object	BE +5.00
			16	Waxy optic discs and thin retinal vessels	BE: 1.2	

Supplementary table 2: Clinical Data

**P10**	3-12mo	Nystagmus<br>Eye poking	17<br>27	Unremarkable<br>Unchanged	BE: NPL<br>BE: NPL	NA
**P11**	3-12mo	Nystagmus<br>Eye poking	<1<br>1	Unremarkable<br>Unchanged	BE: PL<br>BE: PL	BE +4.50/+1.50 x 90°
**P12**	< 3mo	Nystagmus<br>Poor vision	2<br>16	Unremarkable<br>Minimal retinal atrophy	BE: HM<br>BE: CF	RE +6.50/-1.00 x 25°<br>LE +6.50/1.00 x 160°
**P13**	< 3mo	Nystagmus	60<br>79	Peripheral RPE atrophy<br>Pigment hypertrophy with large lacunae of chorioretinal atrophy peripherally<br>Disc pallor<br>Thin vessels<br>Unchanged	BE: HM<br>BE: PL	NA
**P14**	3-12mo	Nystagmus<br>Nyctalopia	1<br>14	Pale discs<br>Thin vessels<br>Fine pigment changes<br>Unchanged	BE: fixate at large object<br>BE: PL	Emmetropia
**P15**	< 3mo	Nystagmus<br>Eye poking	1<br>2	NA<br>Pale disc<br>Otherwise unremarkable	BE: NPL<br>BE: NPL	R +10.00/-2.00 x 30°<br>LE +6.50/1.00 x 160°
**P16**	< 3mo	Poor color vision<br>Nystagmus<br>Photophobia	10<br>19	Unremarkable<br>Unchanged	R: 1.56<br>L: 1.76<br>R: 2.00<br>L: 1.76	R: +1.25<br>L: +1.00
**P17**	< 3mo	Nystagmus<br>Eye poking	1<br>3	Unremarkable<br>Thin vessels<br>Unchanged	BE: PL<br>BE: PL	BE: +8.50
**P18**	< 3mo	Nystagmus<br>Eye poking<br>Photophobia	6<br>10	Blonde fundus<br>Unchanged	BE: PL<br>BE: PL	R: -7.00<br>L: -8.00
**P19**	< 3mo	Nystagmus	8<br>18	Unremarkable<br>Unchanged	BE: PL<br>BE: PL	R: +7.50<br>L: +6.50/+3.00 x 125°

*All patients presented with reduced visual acuity

R; right eye, L; left eye, BE; both eyes, mo; months old, yo; years old, y; years, PL; light perception, NPL = no perception of light; HM = hand motions; NA= not available
